# Prevalence of suicidal ideation among medical students at a university in Brazil

**DOI:** 10.1192/j.eurpsy.2021.1565

**Published:** 2021-08-13

**Authors:** T. Prata, D. Calcides, E. Vasconcelhos, A. Carvalho, E. De Melo, E. Costa

**Affiliations:** 1 Medicine Department, Federal University of Sergipe, Aracaju, Brazil; 2 University Hospital Of The Federal University Of Sergipe, Federal University of Sergipe, Aracaju, Brazil; 3 Medicine Department Lagarto, Federal University of Sergipe, Lagarto, Brazil; 4 Pharmacy Department Lagarto, Federal University of Sergipe, Lagarto, Brazil

**Keywords:** Suicide, Medical Students, mental health, Medical Education

## Abstract

**Introduction:**

Personal and environmental factors may contribute to psychological distress in medical students. As a result, they are more susceptible to suicidal ideation, a serious public health problem.

**Objectives:**

Estimate the prevalence and recognize associated factors of Suicide Ideation and Suicide Attempt among medical students at the Federal University of Sergipe, Brazil.

**Methods:**

A cross-sectional study was performed with randomly selected students between April and June 2019. A structured online questionnaire about sociodemographic characteristics, educational process, and the current psych emotional experiences, besides Beck Scale for Suicide Ideation (BSI), which detects the presence of suicidal ideation, were applied. Statistical evaluation was performed with descriptive analysis and logistic regression for the evaluation of multiple variables.

**Results:**

The study included 133 students, with an average age of 22.9±3.5 and 51,9% were male. Among this sample, 27,1% had suicidal ideation. The frequency is higher in those students who family income <10 minimum wages (OR=3.47) and who were not satisfied with the course (OR=3.52). Furthermore, the frequency of suicide attempt was 15.8%. It was higher among those who claimed to use a doctor-prescribed psychopharmaceutical (OR=10.46) and who lost some discipline in the course (OR=8.17). Ideation and attempt were significantly associated (p<0.001).

**Conclusions:**

Frequency of suicidal ideation was high, associated with dissatisfaction related to the educational process, as well as lower family income. History of attempted suicide was also frequent and associated with ideation. Intervention and prevention measures are required.

